# Sex difference in IRONMAN age group triathletes

**DOI:** 10.1371/journal.pone.0311202

**Published:** 2024-10-07

**Authors:** Beat Knechtle, David Valero, Elias Villiger, Mabliny Thuany, Marilia Santos Andrade, Ivan Cuk, Pantelis T. Nikolaidis, Thomas Rosemann, Katja Weiss

**Affiliations:** 1 Medbase St. Gallen Am Vadianplatz, St. Gallen, Switzerland; 2 Institute of Primary Care, University of Zurich, Zurich, Switzerland; 3 Ultra Sports Science Foundation, Pierre-Benite, France; 4 Department of Physical Education, State University of Para, Pará, Brazil; 5 Department of Physiology, University of Sao Paulo, Sao Paulo, Brazil; 6 Faculty of Sport and Physical Education, University of Belgrade, Belgrade, Serbia; 7 School of Health and Caring Sciences, University of West Attica, Athens, Greece; Geisinger Health System, UNITED STATES OF AMERICA

## Abstract

**Background:**

The sex difference in athletic performance has been thoroughly investigated in single sport disciplines such as swimming, cycling, and running. In contrast, only small samples of long-distance triathlons, such as the IRONMAN^®^ triathlon, have been investigated so far.

**Aim:**

The aim of the study was to examine potential sex differences in the three split disciplines by age groups in 5-year intervals in a very large data set of IRONMAN^®^ age group triathletes.

**Methods:**

Data from 687,696 (553,608 men and 134,088 women) IRONMAN^®^ age group triathletes (in 5-year intervals from 18–24 to 75+ years) finishing successfully between 2002 and 2022 an official IRONMAN^®^ race worldwide were analyzed. The differences in performance between women and men were determined for each split discipline and for the overall race distance.

**Results:**

Most finishers were in the age group 40–44 years. The fastest women were in the age group 25–29 years, and the fastest men were in the age group 30–34 years. For all split disciplines and overall race time, men were always faster than women in all groups. The performance difference between the sexes was more pronounced in cycling compared to swimming and running. From the age group 35–39 years until 60–64 years, the sex differences were nearly identical in swimming and running. For both women and men, the smallest sex difference was least significant in age group 18–24 years for all split disciplines and increased in a U-shaped manner until age group 70–74 years. For age groups 75 years and older, the sex difference decreased in swimming and cycling but increased in running. Considering the different characteristics of the race courses, the smallest performance gaps between men and women were found in river swimming, flat surface cycling and rolling running courses.

**Conclusions:**

The sex difference in the IRONMAN^®^ triathlon was least significant in age group 18–24 years for all split disciplines and increased in a U-shaped manner until age group 70–74 years. For 75 years and older, the sex difference decreased in swimming and cycling but increased in running.

## Introduction

Triathlon is an increasingly popular multi-sport discipline consisting of swimming, cycling, and running, with long-distance triathlon races such as the IRONMAN^®^ Hawaii (3.8 km swimming, 180 km cycling, and 42.195 km running) being the most known [1]. Triathlon represents an excellent model for analyzing the effects of age and sex on endurance performance where the sex differences and the age-related declines in performance can be investigated in the same individuals across the three split disciplines that correspond to three important modes of human locomotion (*i*.*e*., swimming, cycling and running) [2].

It is well known that men are faster than women when competing in endurance disciplines such as swimming [3], cycling [4], and running [5]. The sex difference lies in a range of 10–30% [6]. It is mainly due to differences in anatomy [6] and physiology [7] such as body size [8], limb levers [9], power development [6] and power production [8], muscle mass [8], muscle strength [6] and muscle morphology [10], body fat [8], endurance [6], respiratory-neuromechanical function [10], aerobic capacity [8, 9], oxygen uptake [11], substrate utilization [8, 10] and energetic demands [10], fatigability [12], neuromuscular fatigue [13] and fatigue resistance [10], biomechanics [13], thermoregulation [13], and hormonal control [10]. However, other factors, such as sociocultural, psychological, and sport-specific factors, such as pacing strategies, should also be considered [14].

Recent studies showed, however, that women were able to reduce the gap to men with increasing age in pool swimming for different strokes and distances [15], in long-distance open-water swimming [16], in ultra-cycling in both times- and distance-limited events [17], and in ultra-marathon running [18]. The reduction of the sex difference with increasing age is most likely due to the lower overall participation for both elderly women and men [19] and the higher participation of elderly women [20, 21].

The change in the sex difference in running with increasing age seems to depend upon the participation of women. For example, in ultra-marathon running, the sex difference was more significant when fewer women than men competed [22]. A study investigating more than 1 million of race data from 5 km to ultra-marathon showed that the men-to-women ratio declined with increasing race distance (*i*.*e*., relatively more women competed than men), and the smallest sex difference was reported for runners older than 75 years. Female ultra-marathoners of 75 years and older showed the lowest sex difference of <4%, most likely due to the highly selected population of female ultra-marathoners at this age [21]. Thus, the abovementioned studies indicated that sex differences in performance might depend on the mode of locomotion, age, and race duration.

The aspect of sex difference in the split and overall performance [2] and the interaction of age and sex [23] have been investigated for the IRONMAN^®^ distance triathlon. For IRONMAN^®^ age group triathletes, the sex difference in swimming (~12%) is lower than in cycling (~15%) and running (~18%) [24]. However, most studies were limited to relatively small data sets [25] investigating very narrow samples such as the fastest overall [25] or the fastest age group athletes [2].

The sex difference in split disciplines in triathlon seems to depend upon the discipline [24, 26]. In the IRONMAN^®^ distance triathlon, the sex difference was less significant in swimming compared to cycling and running [23]. For elite triathletes, the sex difference in running performance is greater in the Olympic distance triathlon (~14%) than in the IRONMAN^®^ distance triathlon (~7%) [24]. Also, the magnitude of the sex difference seems to depend on age. In the IRONMAN^®^ distance triathlon, the sex difference increased after 55–60 years [23, 27].

Furthermore, the increase in the sex difference in IRONMAN^®^ triathlon performance with advancing age depends upon the split discipline. For both cycling and running, the sex difference in the performance of the age group athletes older than 60 years was significantly greater than those of younger age groups [27]. For IRONMAN^®^ 70.3 covering the half distance of an IRONMAN^®^ race, the sex difference in performance was lower in swimming compared to cycling and running. Women older than 60 years reduced the gap to men in swimming and cycling, but not in running, where the reduction in the sex difference started after 70 years. The lowest sex difference was in athletes > 75 years old in swimming and cycling and for athletes 30–34 years old in running [28].

In the IRONMAN^®^ triathlon, most finishers are master or age group triathletes [29]. Master athletes are defined as athletes older than 35 years and are divided into categories of 5 years who train and compete in organized teams [30]. Master athletes can perform until very high ages of 80 to 90 years and up to 100–110 years [31]. In recent decades, age group athletes improved their performances in different sports disciplines such as swimming [32], cycling [33], running [34], and triathlon [2]. An important observation is that age group athletes in older age groups seemed to improve in the last decades their performance more than athletes in younger age groups [34].

These findings need verification with a large data set that includes all finishers of all official IRONMAN^®^ races over the last two decades. Based upon this knowledge, the aim of the present study was to investigate the sex differences in split disciplines (*i*.*e*., swimming, cycling, and running) and overall race times by age group in IRONMAN^®^ triathletes. To assemble a large enough sample, we used data from nearly 700,000 women and men IRONMAN^®^ age group race finishes over the last two decades. Based upon existing findings, we hypothesized (i) a measurable sex difference in performance for the three split disciplines and (ii) a decline in the sex difference significance with increasing age, especially in swimming and cycling.

## Method

### Ethical approval

This study was approved by the Institutional Review Board of Kanton St. Gallen, Switzerland, with a waiver of the requirement for informed consent of the participants as the study involved the analysis of publicly available data (EKSG 01/06/2010). The study was conducted in accordance with recognized ethical standards according to the Declaration of Helsinki adopted in 1964 and revised in 2013.

### Data set and data preparation

A dataset with 944,815 records, including professional and age group triathletes’ records, was downloaded from the official IRONMAN^®^ website (www.ironman.com) using a Python script (www.python.org). Each record included the athletes’ full name, sex, age, country of origin, event status, event location and year, the times for swimming, running, cycling, and transition times (represented by transition 1—swimming for cycling, and transition 2—cycling for running) and the full race time (all in seconds). The IRONMAN^®^ website describes a new the possibility that any athlete can compete as a transgender athlete. However, these athletes have to follow special rules (www.ironman.com/news_article/show/1258804). Furthermore, all data regarding name, sex, age, and country of origin are self-reported by the athletes when entering the race.

After isolating the age group records and pre-processing the dataset, a total of 687,696 valid finishers’ records, of which 553,608 were from men and 134,088 from women IRONMAN^®^ age group triathletes, from races between 2002 and 2022 were available for analysis. The athletes’ records were sorted in 5-year age intervals (except the first and last groups) conforming the age groups 18–24, 25–29, 30–34, 35–39, 40–44, 45–49, 50–54, 55–59, 60–64, 65–69, 70–74, 75+. Due to the low numbers of finishers in age groups 75–79, 80–84, and 85–89 years, athletes from these age groups were pooled into an age group of 75+ years. Exclusion criteria were (i) athletes who did not start or finish, (ii) disqualified athletes, (iii) records with missing split time, (iv) records with inconsistent times (*i*.*e*., impossible split times or final times smaller than split times, etc.) and (v) records without essential information (*i*.*e*., sex, age group, etc.). Location data information was added, including average temperatures for air and water and the type of swim (bay, river, ocean, lake, reservoir), bike (flat, rolling, hilly), and run (flat, rolling, hilly) course. This information was merged with the race data, in order to perform analyses of performance with the race course characteristics as covariate.

### Statistical analysis

Trends over the year were calculated and plotted, including participation, men-to-women ratio, and average/ best performance. Histograms of the split and overall race times by sex were plotted, and density distribution curves were overlapped, showing an approximately normal distribution in all cases. Violin plots were used to compare the split and overall race times between age groups and sex, and boxplot charts were used to investigate the performance differences between sex by course types. Detailed data tables were also calculated and included in the study for additional detail, including a large summary table of the different IRONMAN^®^ event locations analyzed through the 20-year period. Statistical significance of the differences between groups was tested through ANOVA two-way and Tukey HSD post-hoc tests. The percent of change between men’s and women’s average times in each age group was also calculated and plotted for each split discipline and overall race times. Descriptive statistics are presented using mean, standard deviation, frequencies, minimum and maximum values, and percentages. The main variables object of study, the full or partial race times, originally recorded in seconds, is converted to hours and further processed with the format HH:MM:SS (hours:minutes:seconds) for presentation in tables. All analysis was done in a Colab Notebook (https://colab.research.google.com/) with Python (www.python.org/) and associated libraries.

## Results

**Fig 1:**
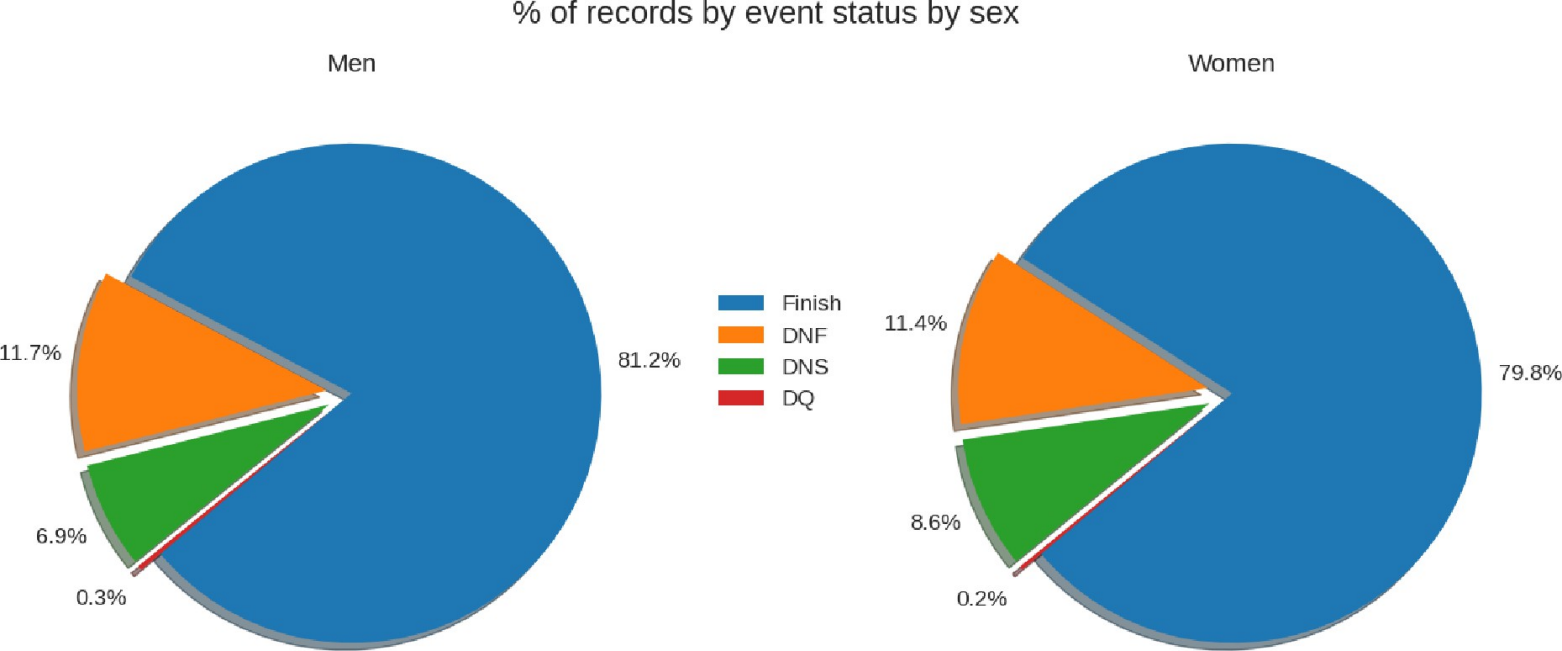
Participants by event status and sex. DNF = did not finish, DNS = did not start, DQ = disqualified.

A total of 687,696 finishers’ records from all official IRONMAN^®^ races held between 2002 and 2022 were analyzed. **Fig 1** shows the breakdown of records in the sample by event status and by sex. Around 20% of all IRONMAN^®^ triathletes who registered to compete did not finish (DNF) the race.

**Fig 2:**
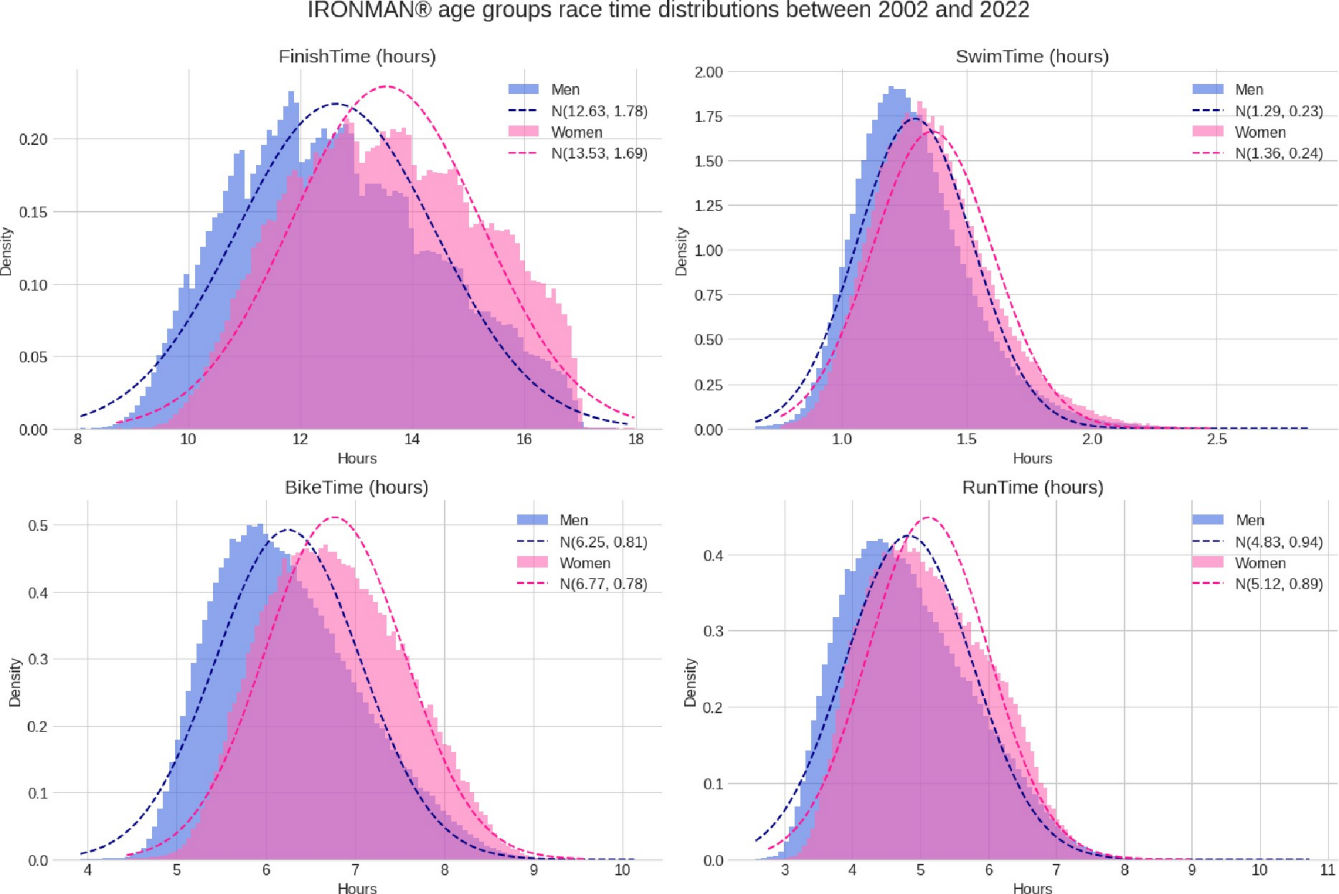
Split and overall IRONMAN^®^ race times histograms for women and men.

**Fig 2** summarizes the mean times for split disciplines and overall race times. Men presented the best finish time and time for each split discipline. Similar values were shown for the highest time (**Table 1**).

**Table 1:** Average split and overall race times (HH:MM:SS).

Men	FinishTime	Swim Time	Bike Time	Run Time
mean	12:37:49	01:17:21	06:14:42	04:50:05
std	01:47:02	00:13:57	00:48:27	00:56:34
max	17:50:33	02:52:23	10:07:28	10:44:04
min	08:03:41	00:39:09	03:55:23	02:34:42
**Women**				
mean	13:31:40	01:21:41	06:46:16	05:07:28
std	01:41:13	00:14:21	00:46:36	00:53:10
max	17:57:34	02:28:29	09:33:28	08:58:57
min	08:42:17	00:45:05	04:26:41	02:45:42

**Fig 3:**
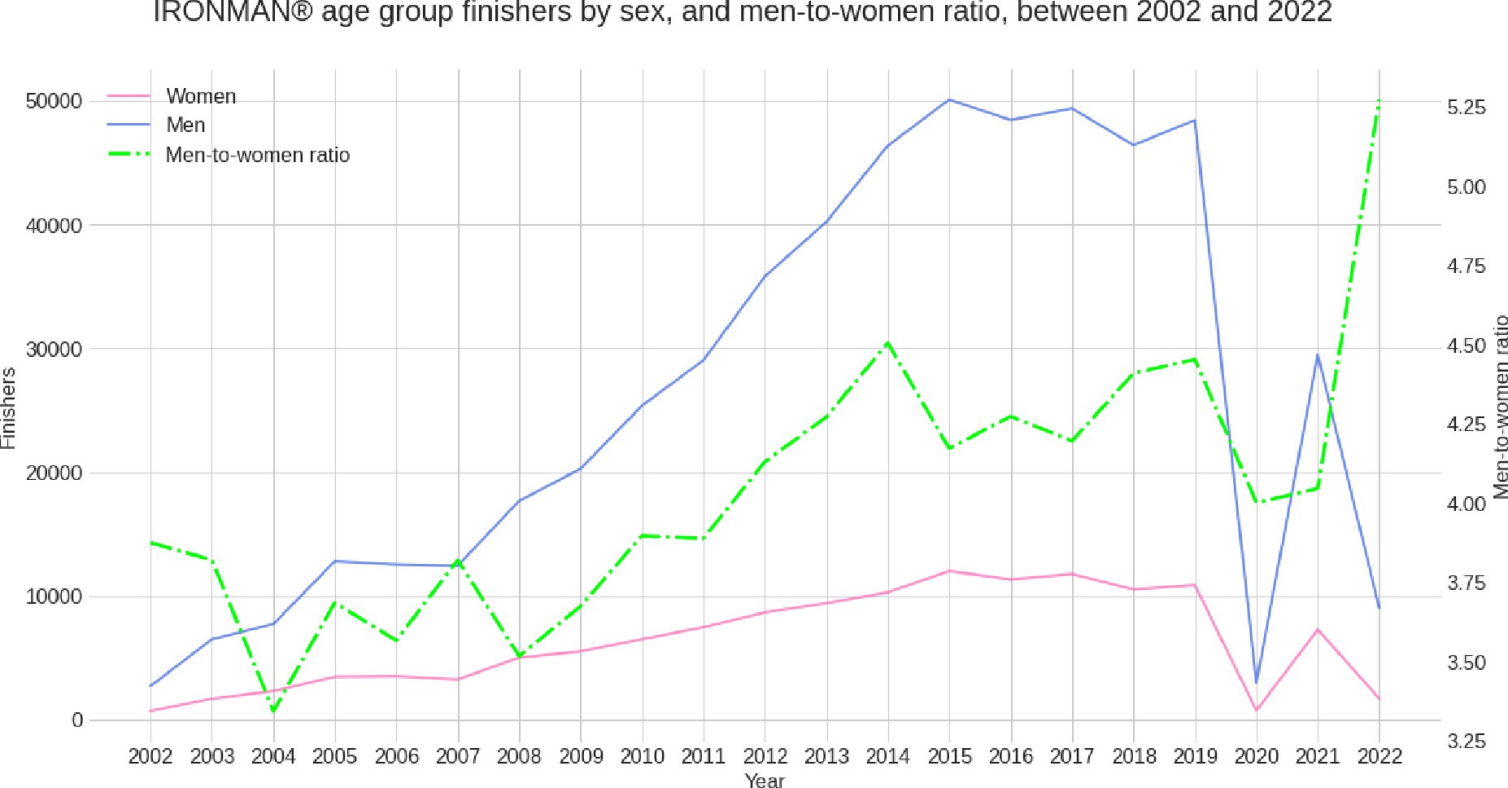
Number of finishers by sex and men-to-women ratio over the years between 2002 and 2022.

**Fig 3** presents the trend in women and men participation over the years with the men-to-women ratio. The number of men finishers increased more than the number of women finishers leading to an increase in the men-to-women ratio over the years. In 2020, the number of finishers dropped dramatically due to the COVID-19 pandemic.

**Fig 4:**
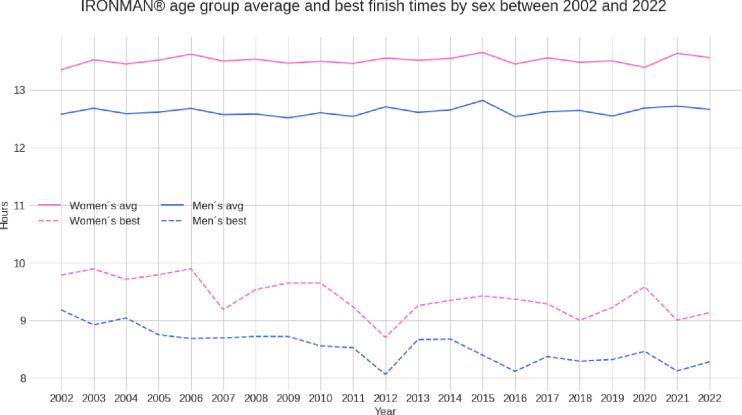
Trends of average and best race times for IRONMAN^®^ age group triathletes.

**Fig 4** presents the trend of overall race times for female and male age groups. Average race times for all age group athletes remained stable during the two decades, whereas the best times of the age group athletes decreased by ~40 min for men and women.

**Fig 5:**
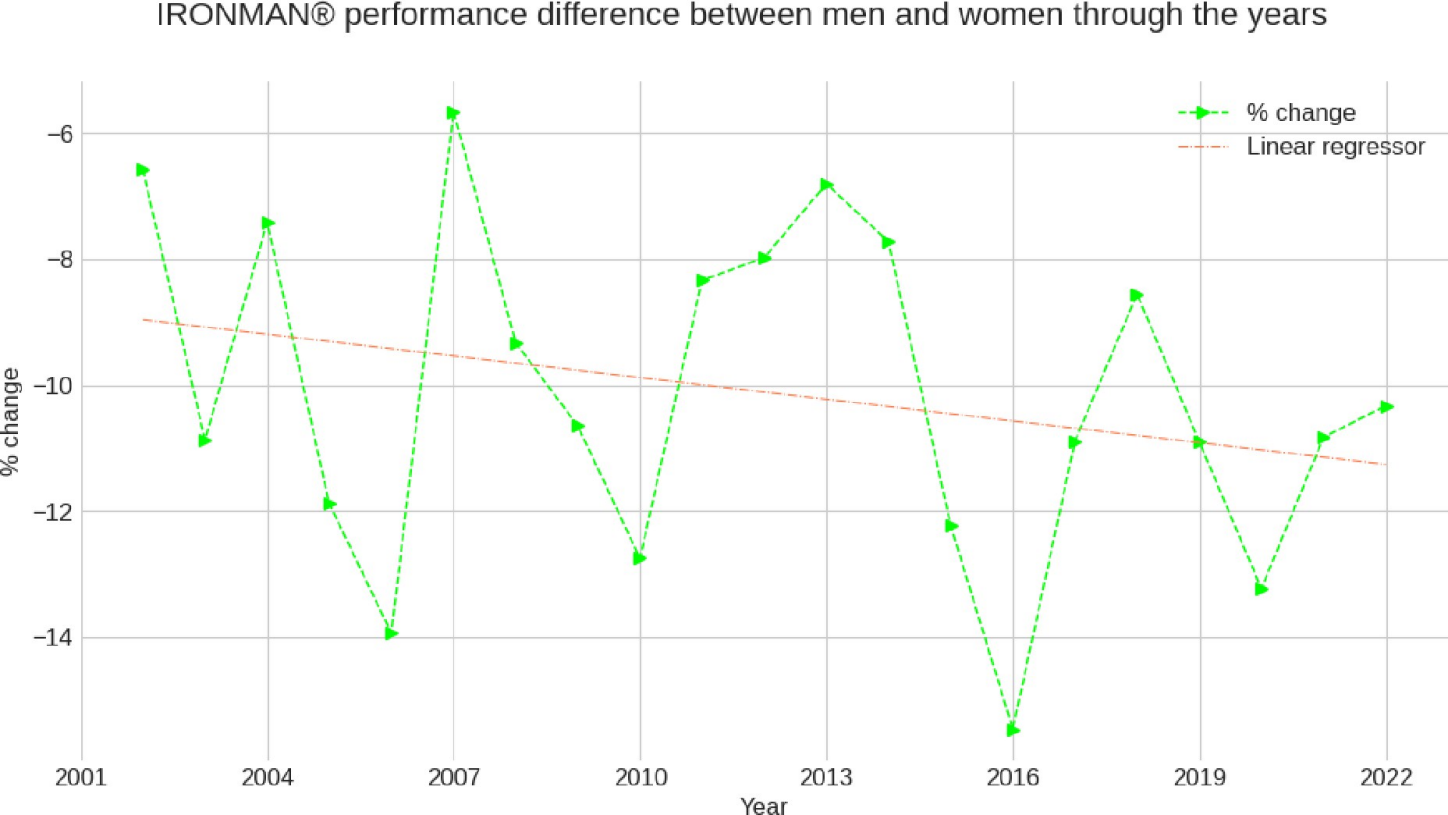
Trend of the sex difference across years in overall race time.

**Fig 5** shows the trend of the overall sex difference across years where an increase in the performance gap through the years of observation seems to have occurred.

**Table 2** summarizes the number of finishers per age group and the corresponding overall race times. For women, most finishers were in age group 40–44 years, whereas the fastest women were in age group 25–29 years. For men, again, most finishers were in age group 40–44 years, but the fastest men were in age group 30–34 years. Overall, race times increased with increasing age.

**Table 2:** Number of finishers by age group for women and men with the overall race times for each age group (HH:MM:SS).

Women overall race times						Men overall race times				
**Age Group**	**n**	**mean**	**std**	**min**	**max**	**n**	**mean**	**std**	**min**	**max**
**18–24**	3409	13:16:04	01:40:08	09:35:50	16:59:42	12931	12:36:07	01:49:36	08:23:48	17:28:31
**25–29**	13568	13:07:35	01:41:06	09:00:49	17:14:54	42310	12:19:28	01:47:18	08:06:57	17:25:48
**30–34**	22689	13:11:43	01:41:47	09:07:09	17:38:06	78919	12:15:21	01:46:16	08:07:22	17:44:01
**35–39**	24647	13:19:36	01:41:19	09:00:00	17:34:20	101705	12:20:21	01:45:19	08:12:43	17:27:31
**40–44**	26505	13:30:14	01:39:32	09:18:57	17:50:43	116384	12:31:19	01:43:48	08:21:39	17:50:33
**45–49**	20848	13:43:29	01:36:48	09:00:08	17:23:49	94384	12:44:21	01:43:01	08:19:20	17:32:48
**50–54**	13757	14:01:25	01:34:16	09:43:47	17:57:34	61987	13:03:32	01:42:17	08:03:41	17:28:07
**55–59**	5853	14:18:42	01:28:18	10:20:58	17:27:28	28367	13:27:46	01:40:35	08:31:11	17:39:05
**60–64**	2106	14:46:36	01:21:46	08:42:17	17:16:58	11413	13:53:40	01:37:04	09:10:50	17:33:02
**65–69**	577	15:17:54	01:07:27	12:02:51	16:59:13	3731	14:21:44	01:29:18	10:04:45	17:14:40
**70–74**	116	15:43:20	00:55:42	13:24:04	16:59:31	1214	14:59:52	01:14:59	08:32:40	17:02:02
**75-**	13	16:33:16	00:39:36	14:41:10	17:02:44	263	15:43:40	01:04:53	08:20:05	17:34:43

**Table 3** shows the men-to-women ratio across the age groups. The ratio increased with increasing age, indicating that more men than women finished with increasing age.

**Table 3:** Men-to-women ratio across age groups.

Age group	men	women	men-to-women ratio
18–24	12,931	3,409	3.79
25–29	42,310	13,568	3.11
30–34	78,919	22,689	3.47
35–39	101,705	24,647	4.12
40–44	116,384	26,505	4.39
45–49	94,384	20,848	4.52
50–54	61,987	13,757	4.50
55–59	28,367	5,853	4.84
60–64	11,413	2,106	5.41
65–69	3,731	577	6.46
70–74	1,214	116	10.46
75-	263	13	20.23

**Fig 6:**
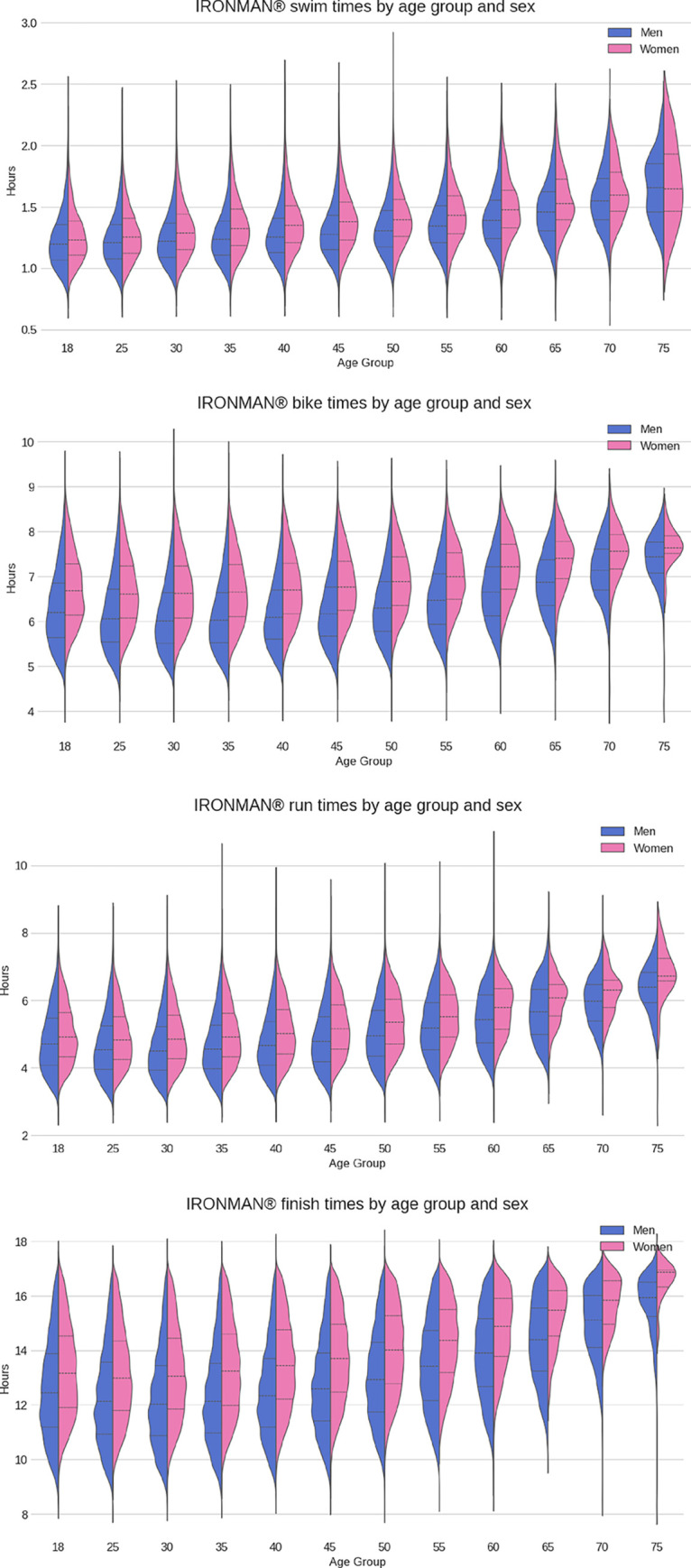
Split and overall race times for women and by age group. Men were always faster than women in each age group (p<0.05).

**Fig 6** presents the split and overall race times by age groups through a set of boxplots. For all split disciplines and overall race times, men were faster than women in all age groups. Cycling times remained flatter than the running and swimming times through the low and mid-age groups, suggesting the cycling discipline exhibits a more consistent performance through the age groups. ANOVA two-way tests were applied to the split and overall race times showing that, for each independent variable (age group and sex) and for their combined effect, the calculated p-values were p<0.0001, and hence we concluded that statistically significant differences existed between age groups and sex.

**Fig 7:**
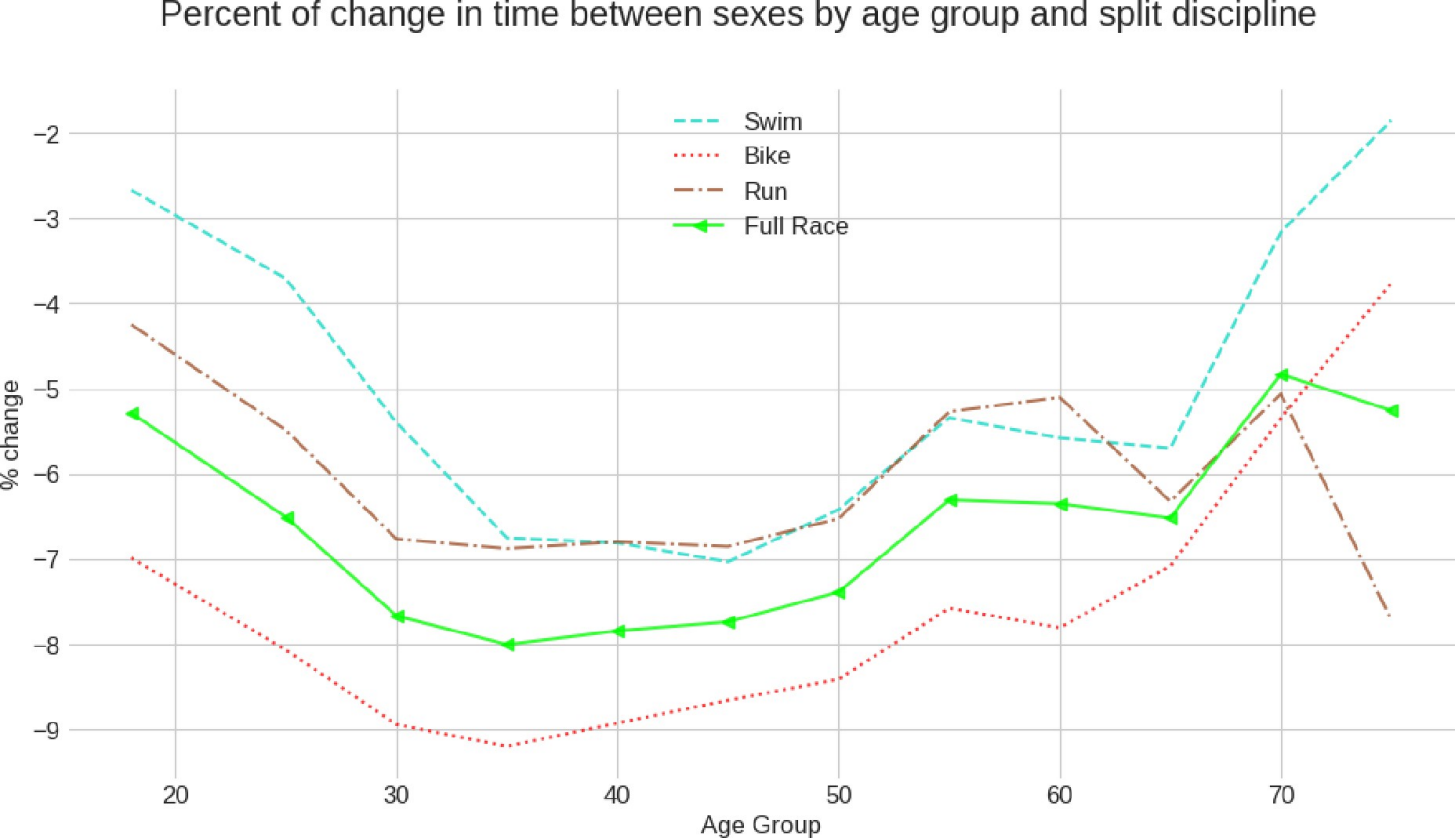
The performance difference between the sexes (in percent) across age groups for split disciplines and overall race times.

**Fig 7** presents the percent change in the time between women and men by age group and split discipline. Men were always faster than women in all age groups and in all split disciplines. The sex difference was more significant in cycling compared to swimming and running. The minimum performance difference was observed in age group 18–24 years for all split disciplines and increased in a U-shaped manner until age group 70–74 years. From age group 35–39 years until 60–64 years, the sex differences were nearly identical in swimming and running. For age groups 75 years and older, we found differences between the split disciplines. The sex difference decreased in swimming and cycling but increased in running.

**Fig 8:**
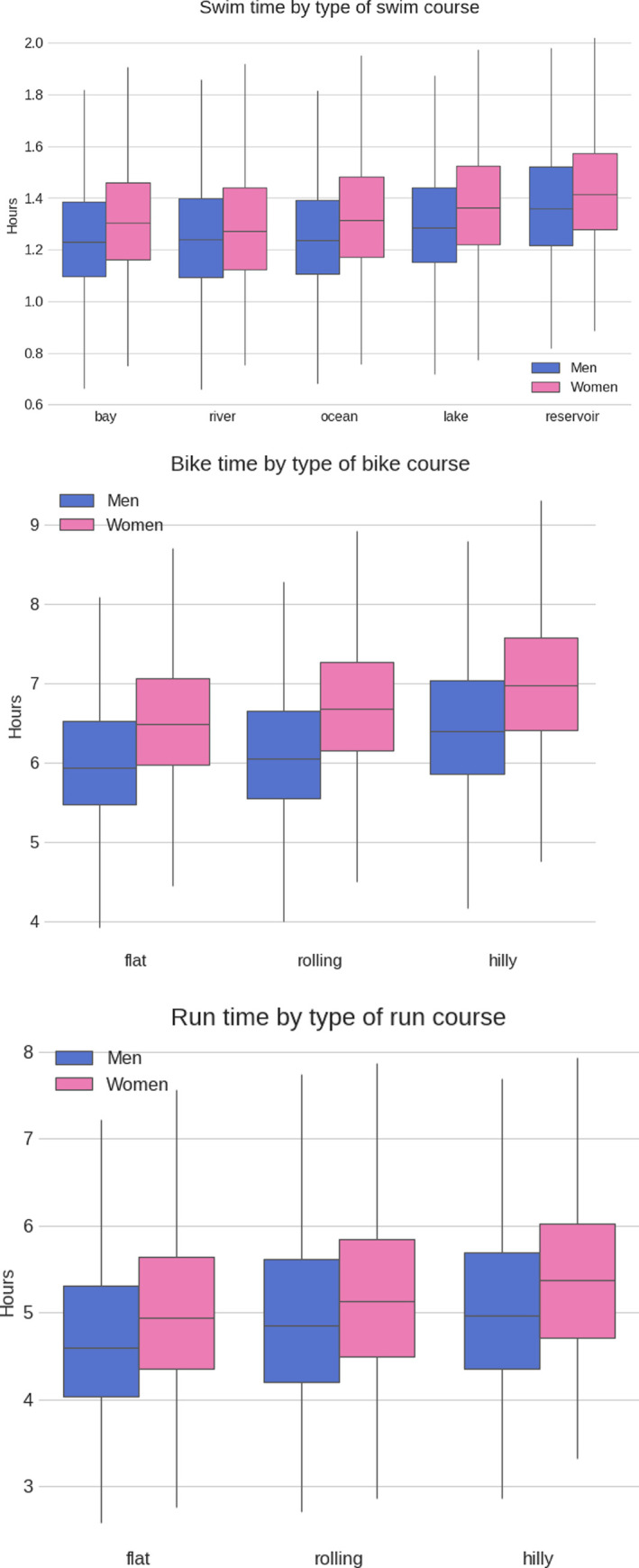
Sex difference in swimming by split disciplines by the type of swim, bike, and run course.

**Fig 8** presents the sex difference by the type of swimming (bay, river, ocean, lake, reservoir), cycling (flat, rolling, hilly), and running (flat, rolling, hilly) course. The smallest performance gaps between men and women can be found in river swimming, flat surface cycling and rolling course running. All the performance differences among women and men and competition courses are statistically significant (p<0.0001).

## Discussion

This study intended to investigate the sex difference in performance with increasing age in IRONMAN^®^ age group triathletes by split disciplines and overall race time. We hypothesized to find (i) a difference in the age-related sex difference by discipline and (ii) a reduction in the sex difference with increasing age, especially in swimming and cycling. We found that (i) men were faster than women in all split disciplines and in all age groups, (ii) the sex difference was more significant in cycling compared to swimming and running, (iii) for both women and men, the sex difference was least significant in age group 18–24 years for all split disciplines and increased in a U-shaped manner until age group 70–74 years (iv) from age group 35–39 years until 60–64 years, the sex differences were nearly identical in swimming and running and (v) for age groups 75 years and older, we found differences between the split disciplines where the sex difference decreased in swimming and cycling but increased in running.

### Men were faster than women

A first important finding was that men were faster than women for all split disciplines and overall races times and for all age groups. Since women were not able to outperform men in the older age groups, we could not definitely confirm our hypotheses that women would close the gap to men in swimming and cycling with increasing age. However, the U-shape indicates a small closing of the gap between women and men at both the low and high ends of the age axis.

A potential explanation for this finding could be the fact that these IRONMAN^®^ age group triathletes were not specialists in swimming and cycling since they must compete in all three disciplines [35]. A further explanation could be that IRONMAN^®^ age group triathletes do not compete in the very old ages, such as master swimmers in pool swimming [15, 36] or master cyclists in ultra-cycling [17]. For pool swimming, it has been shown that women swimmers older than 75 years could achieve a similar performance to men. In master swimmers competing in the FINA World Championships in pool swimming, male age group backstroke swimmers were faster than female swimmers in age groups 25–29 to 80–84 years, but not in age groups 85–89 to 95–99 years [32]. In age group butterfly swimmers, women were not slower compared to men in the master group 90–94 years [15]. In age group breaststroke swimmers, men were not faster than women for age groups 90–94 to 95–99 years [3]. In age group freestyle swimming, women were slower than men in age groups 25–29 to 75–79 years, but not in age groups 80–84 to 85–89 years. In individual medley, men were faster than women from 25–29 to 80–84 years, but not from 85–89 to 90–94 years [36]. And in the FINA World Masters Championships in 3000 m open-water swimming, women were slower in age groups 25–29 to 70–74 years but not in age groups 75–79 and 85–89 years, where race times were similar for both women and men [16].

A subsequent explanation could be the number of women and men finishers in the older age groups. We had only one man in the age group 85–89 years during these two decades, but no women. No athletes older than the age group 85–89 years finished an IRONMAN^®^ triathlon in the last decades. This sex difference in athletic performance is due to physical (*e*.*g*., body size, body composition, length of limbs, running biomechanics), physiological (*e*.*g*., fat mass, muscle mass, muscle tissue characteristics, muscle strength, neuromuscular fatigue, aerobic capacity, oxygen uptake, hormones), technical, thermoregulation, sociocultural, sport-specific, and psychological factors [13, 37, 38]. In ultra endurance swimming, the gap is now less than 5% [8]. Although men have a larger body size with more skeletal muscle mass, a lower percentage of body fat, and a greater maximal delivery of anaerobic and aerobic energy, women have a better capacity to metabolize fat, demonstrate better hydrodynamics, and adopt a more even pacing, which may be advantageous, in particular during long-lasting swimming competitions [8].

### Sex differences in split disciplines

The sex difference in cycling was more significant compared to running, which was more significant compared to swimming. This finding is in contrast to existing reports that describe a less significant sex difference in swimming compared to cycling and running in both the IRONMAN^®^ distance triathlon [23] and the Olympic distance triathlon [24]. A potential explanation could be that our sample consisted of nearly 700’000 race records.

When we consider the single disciplines not performed within a multi-discipline event such as a triathlon, the sex differences were less significant in swimming compared to running [23] and cycling [39]. Similarly, when draft-legal ultra-distance events such as swimming and cycling were compared, the sex difference of the fastest athletes was smaller in swimming than in cycling [40]. In triathlon, the sex difference in split disciplines seemed to depend upon the discipline [41], the age of the athlete [27] and the length of a race [41]. Considering age, the sex difference increased after the age of 35 years in an Olympic distance triathlon [41]. There seem to be differences between Olympic and IRONMAN^®^ distance triathlons where in an IRONMAN^®^ triathlon, the sex difference increased after the age of 55–60 years [23, 27]. Future studies might investigate the physiological, anthropometric, and biomechanical differences between these race distances.

### U-shaped sex difference across age groups

The sex difference was lowest in the age group 18–24 years for all split disciplines and increased in a U-shaped manner until age group 70–74 years. We found that from age group 35–39 years until 60–64 years, the sex differences were nearly identical in swimming and running. The green (run) and light blue (swim) lines overlap between 35 and 55 years completely and hardly vary from 0.5% to 60 years.

One might assume that the U-shaped curve in the sex difference might be due to the distribution of IRONMAN^®^ finishers by age group since athletes in the middle age groups represented most of the finishers and athletes in the younger and older age groups were under-represented. However, the men-to-women ratio increased across age groups indicating that men were overrepresented with increasing age compared to women. Therefore, the sex difference might be due to a selection bias from a lower number of women finishers in the older age groups compared to men finishers. It has been reported that the sex difference in endurance performance regarding age depends upon the participation [22, 41]. A U-shaped sex difference has also been found for age group triathletes competing in Sprint, Olympic, and IRONMAN^®^ 70.3 World Championships. The sex differences for overall race time were greatest in the youngest and older age groups for all three distances [42].

We found differences for the split disciplines after age group 70–74 years, where the sex difference decreased in swimming and running but increased in cycling with increasing age. After the age of 80 years, only 20 men and 1 woman successfully finished an IRONMAN^®^ triathlon. This might have influenced the sex difference in swimming for the age group 75+ years. Regarding the age of 70 years, we also have to consider the age-related performance decline. This performance decline starts at ~30–35 years [43] and remains linear until the age of ~70 years [43], but decreases exponentially after the age of ~70 years [44]. Therefore, from 70–74 years to 75–79 years, this age-related performance decline might explain the differences in the split disciplines. Regarding the age of ~35 years and the widening of the sex difference in performance, we need to consider the maternal age of women and the influence of childbirth occurring often at this age [45]. We also need to consider the impact of 9+ months of limited to no training on performance recover and return to baseline fitness levels following childbirth.

Our findings that the sex difference was lowest in age group 18–24 years might be explained by the low number of finishers and the low men-to-women ratio. However, a study investigating IRONMAN® 70.3 age group triathletes showed that the sex difference in performance was also U-shaped in swimming and running, with an increase after the age of 18–24 years in swimming and after 40–44 years in running. In contrast, the sex difference decreased continuously with increasing age for cycling [28]. Future studies might investigate the potential physiological, anthropometric, and biomechanical differences for this age group compared to older age groups.

### Sex differences in age groups

For age group 75+ years, we found differences between the split disciplines, where the sex difference decreased in swimming and cycling but increased in running. When we consider the age-related performance decline, endurance performance depends upon age [39] and declines with increasing age [39]. For Olympic distance triathletes, the age-related declines in performance were significantly less pronounced for men compared to women for swimming (>50 years), for cycling (>40 years) and for overall race time (>40 years) [26]. A triathletes’ performance decreases in a curvilinear manner with advancing age [2] where the age-related performance decline seems specific to both the split discipline [2] and the race distance [2], where the age-related performance decline differs between race distances [24].

For single disciplines not combined within in multi-disciplines event, we know that women were not able to reduce the gap to men in several different sports disciplines [46]. In recent years, however, women have been able to reduce the gap with men in specific sports disciplines. This has been especially visible for long-distance cycling [47] and swimming of different strokes and distances [15]. In long-distance swimming, women can achieve a similar [16] or even better performance than men [38]. It has been shown that women can beat men in swimming such as long-distance open-water swimming [38].

With increasing age, women seemed to be able to reduce the gap in endurance performance to men [42, 43, 46]. Investigations in swimming, cycling, and running [22, 24, 25] showed that elderly women were able to reduce the gap to elderly men. Specifically, in distance-limited ultra-cycling races covering 100 miles, 200 miles, 400 miles, and 500 miles, the sex difference in cycling speed decreased with increasing age [43]. Also, in time-limited ultra-cycling races of 6 hours, 12 hours and 24 hours in duration, the sex differences in cycling speed decreased between men and women with increasing age [11]. In 50-mile and 100-miles running races, the sex difference decreased with higher ages and was smaller in 100-mile (4.41%) than in 50-mile races (9.13%) [20].

The decrease in the sex difference is most probably because more women compete at older ages [24] and these women are more competitive than men [48]. However, this participation depends upon discipline and race distance. Furthermore, elderly women after the age of 70 years seemed to better preserve muscle quality than men of the same age. It was also assumed that women runners have a greater fatigue resistance than equally trained men. A hormonal influence with advanced age should also be considered [49]. Sex hormones seem to influence differences in the prevalence of diseases, in the magnitude of aging, and in the longevity between men and women [50]. Estrogens have significant impacts on the central nervous system and after menopause, a decrease in estrogens might impact cognitive function [51]. However, towards the end of life, women are frailer and have worse health, while men still perform better [52]. These hormonal changes that occur post-menopausal in women are due to a decrease in estrogen [53]. Furthermore, sex differences do exist regarding musculoskeletal injuries and diseases between women and men at different ages [54].

The sex difference in performance with increasing age seems to depend upon the participation rates to the competitions under analysis. The sex difference in endurance running with increasing age has been described as being dependent upon the participation of women [19]. In ultra-marathon running, the sex difference was more significant when fewer women than men competed [19]. In long-distance running such as marathon [52] and ultra-marathon running [19], the lower participation of women compared to men has an influence on the sex difference in performance.

A further aspect is that we collected data from two decades and that women might have reduced the gap to men over this time. We have calculated and plotted the percent change in overall race time over the years. The finding suggests there may be a slight increase in the performance gap through the years of observation. While we analyzed data from 2002 to 2022, a study investigating race data from IRONMAN^®^ Hawaii from 1983 to 2012 showed that the sex difference for overall race time decreased over years [55]. For the split disciplines, the sex difference remained unchanged for swimming and cycling but decreased for running. Lepers stated in his 2019 review that nowadays, elite female IRONMAN® triathletes are able to reduce the gap with their male counterparts to less than 10% of total performance, thanks to improvement in their marathon running performance [24].

It has been shown that women could reduce the sex difference over years in swimming [25] and in cycling races [11]. The sex difference in running as a weight-bearing discipline seemed to differ compared to cycling and swimming as non-weight-bearing disciplines. The sex gap in running performance is also due to the performance level. The sex difference was least significant between women and men world records in running but increased in lower-ranked athletes [14]. We also need to consider that there exist sex inequalities [56] and training and coaching differences for female athletes on their way.

### The aspect of distance

The reduction of the sex gap seemed to depend upon the length of an endurance performance. In running, the sex difference increased with increasing running distance [17]. In running races from 60 m to 10,000 m, the sex difference increased over distances [57]. For longer distances such as a half-marathon and a marathon, the sex difference was less significant for the marathon distance compared to the half-marathon [58]. In half-marathon and marathon running, the sex difference in performance was less significant in the longer race distances and the older age groups [58]. When 12- and 24-hour running races were compared, the 24-hour race showed a lower sex difference in performance [33]. This might also be due to participation differences. In ultra-cycling races from 6 hours to 24 hours, the sex differences in cycling speed decreased with increasing duration of ultra-cycling races [17]. In races from 100 miles to 500 miles, no sex difference was identified for 400 miles and 500 miles [33]. When trail runners of the same performance level were investigated, the gap between women and men became reduced with increasing running distance, demonstrating that endurance is greater in women compared to men [59]. In triathlon races, the sex difference increased from the IRONMAN^®^ distance from IRONMAN^®^ Hawaii to the double distance of the Double Iron ultra-triathlon [60] and the Deca Iron ultra-triathlon [48].

### The aspect of participation and performance level

The men-to-women ratio in long-distance events seems important for the sex difference. In running races from 5 km to ultra-marathon covering a data set from more than 1,100,000 running race records, women were more prevalent in shorter races (*i*.*e*., 5, 10 km, half-marathon) and outnumbered men in 5 km races. With increasing race distance, the men-to-women ratio declined, and the sex difference decreased until 70 years of age, after which it varied depending on the race distance. Elderly female ultra-marathoners (*i*.*e*., 75 years and older) displayed a performance difference of <4% compared to male ultra-marathoners, most likely attributed to the presence of highly selected outstanding female performers [21]. The reduction of the sex difference with increasing age is most likely due to the lower overall participation [20] and the higher participation of elderly women [20] leading to a selection of the best female ultra-endurance athletes in the higher ages [21]. When female and male ultra-marathoners with comparable numbers in 50-mile and 100-mile ultra-marathons were compared, the sex difference was reduced to 1%-3% [61]. We also need to consider the aspect of performance level. In running events from 100 m to the marathon, the sex difference varies with the performance level. The difference in absolute running performance between men and women is lowest for world record/world lead performances and increases in lower-ranked elite athletes [14].

### Limitations

Some limitations must be acknowledged regarding the variation of race characteristics across different locations (*e*.*g*., environmental conditions) and the advancement of technology during the last two decades. The data stems from different race locations where the distances of the split disciplines might not have been exactly measured. Furthermore, during the two decades, the technologies in cycling (*i*.*e*., time trial bikes) might have advanced considerably. The influence of environmental conditions and changes in altitudes were not considered. Also, caution is needed when interpreting the findings on the old age groups, where the number of women (12 in the age group 75–79 years and one in the 80–84 years age group) would not allow safe conclusions. We have to acknowledge the limitation of the men-to-women ratio for a comparative analysis. With a men-to-women ration between 3 and 4.5 we do not believe however the male over-representation to be excessive, but just a reflection of the competition demographics. A further limitation is that post-partum information was not collected nor accounted for in data analysis. And a last limitation was that we have no data about transgender athletes. Transgender athletes are considered in this study as the sex identify with at the time of the racing. On the other hand, this cross-sectional data analysis uses the most extensive data set to date regarding the analysis of the sex difference in IRONMAN^®^ age group triathletes. Another strength of the present study was that it used the model of triathlon to examine sex differences in three basic modes of human locomotion, and this methodological approach was better than separate swimming, cycling, and running races of different athletes.

## Conclusions

In summary, men IRONMAN^®^ triathletes were faster than women IRONMAN^®^ triathletes in all age groups and in all three split disciplines. The race time difference between sexes in cycling was more significant compared to swimming and running in all age groups. The difference was the least significant in 18–24 years for all split disciplines and increased in a U-shaped manner until 70–74 years. For age groups 75 years and older, the sex difference decreased in swimming and cycling, but increased in running.

## Supporting information

S1 Checklist Human participants research checklist. (DOCX)

S1 Data (XLSX)

S2 Data (XLSX)

## Abbreviations

ANOVA: Analysis of variance
DNF: Did Not Finish
DNS: Did Not Start
DQ: Disqualified
